# Getting the Right Evidence After Drug Approval

**DOI:** 10.3389/fphar.2020.569535

**Published:** 2020-09-09

**Authors:** Rick A. Vreman, Hubert G. M. Leufkens, Aaron S. Kesselheim

**Affiliations:** ^1^ Division of Pharmacoepidemiology and Clinical Pharmacology, Utrecht Institute for Pharmaceutical Sciences, Utrecht University, Utrecht, Netherlands; ^2^ National Health Care Institute (ZIN), Diemen, Netherlands; ^3^ Program on Regulation, Therapeutics and Law (PORTAL), Division of Pharmacoepidemiology and Pharmacoeconomics, Department of Medicine, Brigham and Women’s Hospital/Harvard Medical School, Boston, MA, United States

**Keywords:** evidence, comparative evidence, regulation, pricing, value-based, health technology assessment, reimbursement

## Abstract

To generate comparative evidence in a timely fashion for drugs without restricting or delaying access, value-based pricing and reimbursement could be conditioned on a prospective, post-approval evidence generation plan.

## Introduction

Drug regulatory policies aim to balance evidence generation with patient access. Overly onerous demands for evidence lead to longer development times and higher costs. Approving drugs too early may lead to uninformed use due to unestablished or uncertain benefits and risks, potentially harming patients and adding to increased and unjustified health care spending (Eichler et al., 2018).

## Drug Approval Versus Clinical Practice

After a drug is approved, a key issue for patients, prescribers, and payers is how much clinical benefit on patient-relevant outcomes can be expected compared to available alternatives. However, formal regulatory requirements by the FDA or EMA do not include active control comparisons based on patient-relevant clinical endpoints, resulting in a limited number of regulatory dossiers containing such data (Kim and Prasad, 2015; Hatswell et al., 2016; Vivot et al., 2017; Morant and Vestergaard, 2018; Chen et al., 2019; Naci et al., 2019). Furthermore, drugs may have variable comparative effects in different subgroups of the patient population, which often remain unstudied or not captured in the label.

A lack of comparative evidence may be particularly evident for drugs approved *via* expedited pathways such as accelerated approval in the United States or conditional approval in Europe. Both regulatory systems require under such circumstances additional evidence generation post-approval for these drugs, but studies have revealed that these post-approval trials often have similar characteristics to pre-approval trials, and are too frequently delayed or difficult to get finished (Hoekman et al., 2016; Naci et al., 2017; Bloem et al., 2019). After drugs are approved and reimbursed, manufacturers have little incentive to perform additional active control trials within the same indication. Indeed, 81% of industry initiated non-obligated post-approval trials were to study new indications or indication expansions rather than approved indications (Skydel et al., 2019).

Lack of comparative evidence can complicate treatments decisions. For example, in chronic lymphocytic leukemia, treatment choices can be based on genetic abnormalities, fitness of patients, previous treatments and timing of relapse, among other factors. The myriad of newly approved targeted treatment options provide a promising outlook for patients with this condition (Yeung and Shadman, 2019). However, a recent review of available treatment options for chronic lymphocytic leukemia also emphasizes that the current available evidence leaves many questions unanswered because of single-arm trial designs and a lack of robust data on active comparisons, on combination regimens, on long-term outcomes, and on treatment effects in subpopulations (Yeung and Shadman, 2019).

## Why Are There So Many Unanswered Questions?

It is perhaps unsurprising that current regulatory standards lead to many remaining unanswered questions at the time of drug approval. A positive benefit-risk balance as assessed by regulators does not necessarily provide answers to questions on added therapeutic benefit that may be of most interest to clinicians and payers (Eichler et al., 2019). Additionally, indications can be granted on average effects without sufficient power to draw conclusions for subpopulations.

If questions about comparative clinical effects in relevant subgroups at approval are only partly addressed by post-approval trials obligated by regulators, how can we get the evidence patients, clinicians and payers need?

One pathway may be to leverage organizations that conduct health technology assessment (HTA), a term that encompasses clinical and economic value assessments of new drugs informed by the evidence and medical experts. Independent organizations in the United States such as the Institute for Clinical and Economic Review (ICER) systematically assess comparative benefits on patient-relevant endpoints of certain drugs. ICER explicitly highlights remaining uncertainties related to the available evidence beyond the benefit-risk balance. In Europe, formal HTA processes exist in most countries, leading to dossiers about comparative benefit and economic value of medicines similar to those of ICER. However, ICER has no mandate to enforce evidence generation and in Europe additional evidence generation for HTA purposes is organized nationally with only little international coordination.

Besides outlining the remaining uncertainties and the types of trials needed to resolve them, HTA processes can also provide suggestions for reasonable drug prices based on the drug’s effects and the uncertainties around these effects. Linking the value that a product delivers to its price may incentivize the development of transformative drugs. Value-based pricing mechanisms have contributed to lower drug prices in Europe as compared to the United States (Kang et al., 2020). However, lowering prices for drugs for which clinically relevant comparative benefits are still unclear does not solve the problem that patients and clinicians face when they must decide which treatment to start. Ultimately, it must be established whether a drug provides meaningful benefits over alternative treatment options.

Clearly, there are institutes such as HTA organizations that have the capacity to outline the most relevant uncertainties to patients, clinicians, and payers that should be resolved post-approval. But who can enforce this type of evidence generation?

## Promoting Post-Approval Evidence Generation

To increase post-approval evidence generation, regulators could be given more authority to work with HTA bodies to determine an agenda for post-approval comparative trials related to newly-approved drugs. In a comprehensive review of regulatory and HTA dossiers, we found that regulators and HTA bodies were usually able to agree on appropriate endpoints and comparators in pre-approval scientific advice procedures, and that manufacturers often followed this combined advice for clinical trials (Tafuri et al., 2016). Such joint efforts do not yet exist for post-approval trials, and through this pathway, remaining uncertainties related to comparative clinical effects within the relevant patient populations can be identified at approval.

However, studies have pointed to regulators’ inability to consistently enforce post-approval trial obligations, because it is politically and socially challenging to withdraw marketing authorization purely because evidence generation is delayed. In a previous study, seven guiding principles were put forward to guide the generation of evidence post-approval. One of the principles states that it is necessary to create the right incentives for generating comparative evidence post-approval (Cipriani et al., 2020). One way to do this would be to make drug prices conditional upon resolving the most important uncertainties, leading to price premiums for more certainty and price penalties for delayed evidence generation. Such a prospective approach can facilitate fair return on investment for post-approval evidence generation for manufacturers while simultaneously reducing financial uncertainty for payers.

The viability of such an approach depends on the willingness of the involved stakeholders. In the US, private insurers already negotiate drug prices and they have stressed the relevance of value assessments for coverage and pricing decisions (Augustovski and McClellan, 2019). However, there is no governmental HTA institute and there is also no formal coordination between the FDA and non-governmental HTA institutes. In Europe, results of coverage with evidence development schemes are mixed and a central approach that is aligned with the EMA is lacking. The European Network for HTA (EUnetHTA) may facilitate the coordination between the EMA and national HTA institutes. Most vital is that price incentives facilitate research post-approval that is not duplicative, answers the most important uncertainties for all stakeholders, and is done in the most efficient and timely way.

## Conclusion

Clinical evidence at drug approval is often not sufficient to ensure informed clinical and reimbursement decision making. One solution to incentivize companies to generate such comparative evidence in a timely fashion without restricting or delaying access could be to condition value-based pricing and reimbursement on a prospective, post-approval evidence generation plan. Whether such schemes will deliver on their promise depends on the quality of early dialogue between stakeholders, predictability of the outcomes, and on stakeholders’ ability to establish a fair balance between sufficient prospects of returns on trial investments for manufacturers and reasonable value for money for payers.

## Author's Note

HGML declares that he is a member of the Lygature Leadership Team.

## Author Contributions

All authors contributed to the article and approved the submitted version.

## Funding

HL declares that he is a member of the Lygature Leadership Team. AK’s work is supported by Arnold Ventures, as well as the Harvard-MIT Center for Regulatory Science. The funders were not involved in the study design, collection, analysis, interpretation of data, the writing of this article or the decision to submit it for publication.

## Disclaimer

The views expressed in this article are the personal views of the authors and may not be understood or quoted as being made on behalf of or reflecting the position of the agencies or organizations with which the authors are affiliated.

## Conflict of Interest

The authors declare that the research was conducted in the absence of any commercial or financial relationships that could be construed as a potential conflict of interest.
